# The positive valence system, adaptive behaviour and the origins of reward

**DOI:** 10.1042/ETLS20220007

**Published:** 2022-11-14

**Authors:** Thomas J. Burton, Bernard W. Balleine

**Affiliations:** Decision Neuroscience Lab, UNSW Sydney, Botany Street, Randwick, NSW 2052, Australia

**Keywords:** arousal, evaluative conditioning, habit, instrumental conditioning, motivation, Pavlovian conditioning

## Abstract

Although the hey-day of motivation as an area of study is long past, the issues with which motivational theorists grappled have not grown less important: i.e. the development of deterministic explanations for the particular tuning of the nervous system to specific changes in the internal and external environment and the organisation of adaptive behavioural responses to those changes. Here, we briefly elaborate these issues in describing the structure and function of the ‘positive valence system’. We describe the origins of adaptive behaviour in an ascending arousal system, sensitive to peripheral regulatory changes, that modulates and activates various central motivational states. Associations between these motivational states and sensory inputs underlie evaluative conditioning and generate the representation of the ‘unconditioned’ stimuli fundamental to Pavlovian conditioning. As a consequence, associations with these stimuli can generate Pavlovian conditioned responses through the motivational control of stimulus event associations with sensory and affective components of the valence system to elicit conditioned orienting, consummatory and preparatory responses, particularly the affective responses reflecting Pavlovian excitation and inhibition, arousal and reinforcement, the latter used to control the formation of habits. These affective processes also provoke emotional responses, allowing the externalisation of positive valence in hedonic experience to generate the goal or reward values that mediate goal-directed action. Together these processes form the positive valence system, ensure the maintenance of adaptive behaviour and, through the association of sensory events and emotional responses through consummatory experience, provide the origins of reward.

## Introduction

The term ‘positive valence system’ has been defined descriptively within the National Institute of Health's Research Domain Criteria (RDoC) as ‘the collection of processes that produce responses to positive motivational situations or contexts,'^1^
^1^https://www.nimh.nih.gov/research/research-funded-by-nimh/rdoc/positive-valence-systems-workshop-proceedings i.e. consummatory responses, preparatory responses, goal-directed actions or habits. Here, we briefly elaborate current evidence regarding the structural processes productive of these forms of behavioural response and how, structurally, these processes are integrated to generate a functional ‘positive valence system’ capable of maintaining adaptive behaviour.

It is important, however, to carefully unpack what apparently innocent terms like ‘positive motivational situations and contexts' should be taken to mean. When considering the nature of motivational control, it is necessary carefully to differentiate structure from function; i.e. the structure of any motivational or ‘valence’ system from the influence that system has on behaviour. An unconditioned response (UR) — such as an approach response elicited by an unconditioned stimulus — can appear more or less identical with a conditioned response (CR), a goal-directed action or a habit. However, the structure controlling each of these types of response differs considerably. In evaluating the broader system of motivational control, it is the discovery of its structural components and how they are integrated that allows us to understand its behavioural effects including, in this case, those concerned with positive valence. In what follows we briefly explore the structure of the positive valence system, its relationship to adaptive behaviour and to reward learning. For clarity we have collected some of the more unfamiliar terms in this literature in a glossary — see Box 1.

**Box 1. Glossary of terms**

*Appetitive–aversive interaction*: The interaction of an appetitive CS with aversive CS or vice versa.

*Blocking*: The finding that a CS previously paired with a US reduces conditioning to another CS when they are conditioned in compound.

*Central motivational state (cms)*: A hypothetical entity that converts drive and drive stimuli into incentive values.

*Consummatory responses*: Discrete behavioural reactions to specific incentive events, e.g. chewing, licking, etc.

*Conditioned appetitive excitor*: A stimulus that predicts an appetitive event, e.g. a tone that predicts food.

*Conditioned appetitive inhibitor*: A stimulus that predicts the absence of an appetitive event e.g. a light that predicts no food.

*Conditioned taste preference/aversion*: A procedure in which a taste is paired with positive/negative post-ingestive consequences.

*Counterconditioning*: Pairing a CS that was previously paired with an appetitive event with an aversive one or vice versa.

*Drive*: A hypothetical construct that accounts for the increase in performance induced by an increase in motivation.

*Evaluative conditioning*: The effect of pairing specific sensory events with a central motivational state or *cms.*

*Goal-directed actions*: Actions controlled by the encoding of their causal consequences and the value of those consequences.

*Habitual actions*: Actions that, through reinforcement, have become associated with, and are elicited by, specific stimuli.

*Homeostasis*: A regulatory process that maintains internal bodily states at a relatively stable equilibrium.

*Positive Incentive*: Any stimulus or event towards which a subject is attracted.

*Irrelevant incentive effects*: Latent learning about the incentive properties of an event irrelevant to the prevailing motivational state.

*Instrumental conditioning*: A procedure in which it is arranged that an action is instrumental to the occurrence of a specific outcome.

*Latent inhibition*: The finding that preexposure to a conditioned stimulus slows learning of its association with a US.

*Liking*: The hedonic response elicited by exposure to an event resulting in changes in the instrumental incentive value of that event.

*Pavlovian conditioning*: A procedure in which a stimulus is associated with one another, usually biologically significant, stimulus.

*Pavlovian-instrumental transfer*: A procedure assessing the effect of a CS on instrumental performance.

*Preparatory responses*: Diffuse behavioural reactions to predictive stimuli such as general search, approach or withdrawal.

*Reinforcement*: Feedback generated by the instrumental outcome that strengthens stimulus–response associations.

*Reward*: Feedback generated by the instrumental outcome that strengthens action-outcome associations.

*Second-order conditioning*: A change in the response to a stimulus when it is paired with a stimulus previously associated with a US.

*Sensory preconditioning*: A change in the response to a stimulus previously paired with a stimulus subsequently associated with a US.

*Transreinforcer blocking*: The preservation of blocking despite a change in the US identity across the phases of a blocking design.

*Wanting*: A conditioned preparatory response elicited by a CS reflecting the incentive motivation induced by that stimulus.

## Evaluative conditioned responses

**Figure 1.**
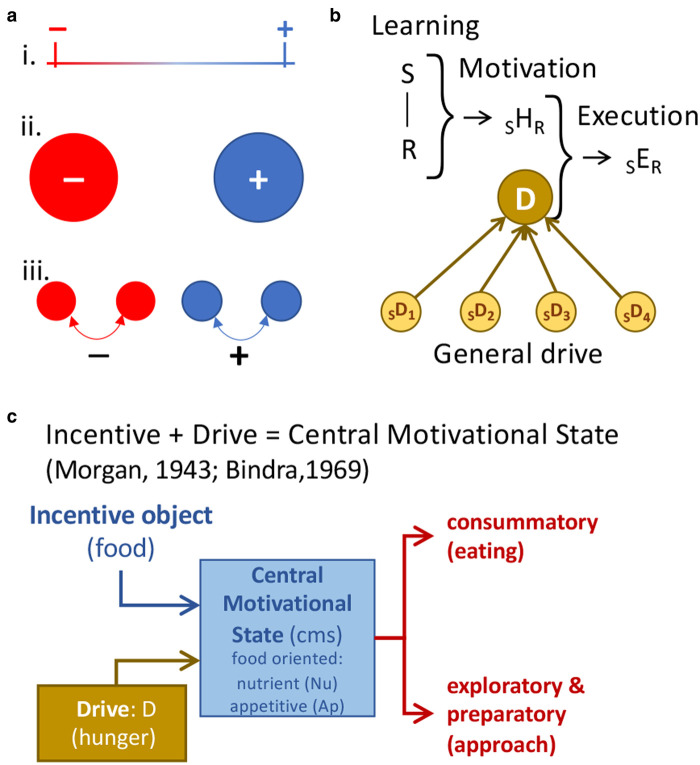
Valence, drive and the central motivational state. (**a**) Various forms of valence process: (i) monovalent continuum; (ii) bivalent system composed of a positive and negative state; (iii) a multi-nodal valence network with valence emerging from the interaction of multiple processes. (**b**) Hull's general drive theory: S–R learning reinforced by drive reduction generates in habit strength (_S_*H*_R_) which, multiplied by drive, results in response execution (_S_*H*_R _× *D* = _S_*E*_R_). *D* = drive and _S_*D*_n_ = sources of drive. (**c**) Incentive theories that incorporated drive provided the basis for Central Motive State accounts of motivated behaviour.

It is a non-trivial task to establish the nature of valency [1]. Should it be regarded as: a single continuous process, with negative and positive valence emerging only at the extremes of a continuum; a bivalent system composed of a positive valence system opposing, say, one concerned with negative valence; or a multivalent system with positive and negative valence emerging from the interaction of multiple component processes involved individually in specific features of motivated behaviour (Figure 1a)? Nor is it entirely clear whether valence should be regarded as a quantitative construct or as one synonymous with specific affective or emotional states. Here the ‘positive’ could be interpreted variously as an energetic condition, something adaptively beneficial, positive or emotionally pleasant. There are data consistent with all these perspectives.

Historically, motivational analyses were aligned purely with energetic constructs such as homeostasis or drive [2,3], the most thorough-going account of which was Hull's theory of general drive [4]. On this view, valence is linked to the operation of a single drive and instantiated quantitatively on a continuum. Motivated behaviour is released by an increment in drive in the service of returning to drive homeostasis, with such increases generating a negative valence and reductions in drive a positive valence. Reductions in the drive were argued to produce positive reinforcement and to strengthen stimulus–response (S–R) associations and increases in the drive to weaken them, allowing internal and external stimuli to acquire the capacity to provoke specific adaptive responses that ameliorated certain needs or threats. Importantly, rather than multiple independent drives (such as hunger, thirst, sex), Hull proposed a single ‘general’ drive to which multiple sources of drive contribute, the implication being that, regardless of valence, sources of drive could both substitute for and summate with one another. This became an important testing ground for the theory. Some early evidence provided support; for example, up to a point, responses established with food deprivation could be maintained (‘substituted’) or incremented (‘summated’) by water deprivation and even with mild footshock [5,6]. However, beyond that point substitution and summation collapsed.

**Figure 2.**
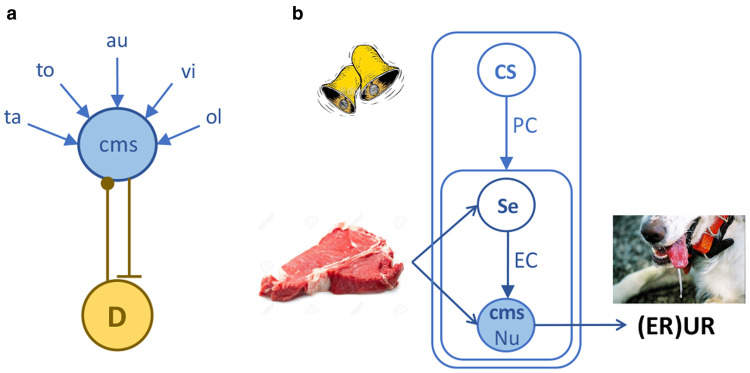
Evaluative conditioning and its role in and Pavlovian conditioning. (**a**) Evaluative conditioning as the association of sensory features with a central motivational state (cms). The activity of the cms is modulated by drive and feedback to regulate that modulation; e.g. an increase in food deprivation (increasing drive) lowers the threshold to detect nutrients, the sensing of which feeds back to reduce the influence of food deprivation on that threshold. NB: ta = taste; to = touch; au = auditory; vi = visual; ol = olfactory features of sensory events. (**b**) The relationship of Pavlovian conditioning (PC) and evaluative conditioning (EC). An event (meat) productive of an unconditioned response (UR, salivation) is not immediately represented but is learned through sensory event (Se) associations with a central motivational state sensitive to nutrients (cms — Nu). As a consequence, those sensory events, e.g. the sight of meat, can evoke an evaluative response (ER, salivation). Neutral events, e.g. a bell, can now also be associated with the ‘US’ and evoke other conditioned responses (see Figure 3).

Around the same time, Pavlov's ‘Conditioned Reflexes’ was published in the west [7] and although it was not a motivational treatise, it provided an important impetus to such accounts by focusing attention on the origin and elaboration of motivated behaviour, mostly digestive reflexes, in the production of UR and CR to positive and negative events [8]. Importantly, Pavlov defined unconditioned stimuli (USs) in terms of the responses they evoked and not their representation. He described the consummatory responses evoked by food in hungry dogs on first contact and the subsequent generalisation of those responses to the sight and smell of the food itself, arguing the latter reflects a process of what he called *signalisation.* These observations formed the core of various incentive theories of motivation (Figure 1c) that proposed that, rather than being solely dependent on the internal drive, it is the anticipation of positive, affectively appetitive events that provide the proximal source of motivated behaviour [9–12]. These views were elaborated structurally to incorporate drive into a ‘drive + incentive’ perspective referred to, historically, as a *central motivational state* (*cms*) [13,14]. From this perspective, signalization is essentially a process of initial evaluation generating evaluative incentives — Figure 2a; i.e. it is a process engaged during the early experience with an event where the sensory properties of the experience (e.g. the taste of a particular food) are integrated with a central motivational state sensitive to nutritive events. In Pavlovian terms, this process generates a representation of what would ordinarily be described as a US; Figure 2b. Specific foods, fluids, sources of warmth can evoke URs but as evaluative incentives they can also become events (or stimuli) about which animals can learn; i.e. they can become associated with other events. As a consequence, such stimuli can come to generate conditioned responses of various forms, not just because they reduced sources of drive induced by hunger, thirst or thermoregulatory needs, but because of their motivational, and affective consequences.

Moll [15], for example, describes findings consistent with Pavlov's ‘signalization’ process in young rats. On their first experience with food deprivation the rats ate substantially less than was required to overcome their deficit or even to maintain their nutritional needs, although they rapidly learned to increase consumption over time and over presentations of the food. Similarly, Changizi et al. [16] found that rat pups did not show food-seeking behaviour when food deprived unless they had previous experience with food under food deprivation. Perhaps more surprisingly, they also reported evidence that the same is true of water for thirsty rats [16,17]. Furthermore, the experimental procedures used to generate evaluative incentives have obvious similarities to those used to generate conditioned taste preferences (and aversions); i.e. rats are generally first deprived of some essential commodity (e.g. nutrients, fluids, or, more specifically, sodium or calcium, etc.) after which a stimulus (usually a taste) is paired with the delivery of the deprived commodity (whether in solution or via intragastric, intraduodenal, hepatic portal or intravenous routes) [18]. As a consequence, rats significantly increase their willingness to contact and consume the taste itself relative to those given the taste and commodity unpaired [19]. Such treatments also increase ingestive, orofacial responses when the paired taste alone is contacted [20]. These responses depend on deprivation [21]; i.e. on the visceral and humoral signals originating in regulatory processes such as those that control feeding, drinking and so on [22] (Figure 2). Although not the focus here, Garcia [23] has argued that conditioned taste aversions too, are best viewed as an example of evaluative conditioning (what he called ‘Darwinian conditioning’).

Evaluative responses reveal, therefore, the basic foundation of incentive learning, whereby the sensory-specific properties of a motivationally significant event, or US, come to generate the specific consummatory reflexes that were initially only elicited by contact with the US itself. This requires the formation of associations between specific sensory features of those events and central motivational states, with the latter being dynamically modulated by primary drive states such as hunger. But how does a valence structure support behaviour that is of more general consequence for reward acquisition?

## Pavlovian conditioned responses

Accounts of Pavlovian conditioning usually emphasise the association between stimulus representations rather than between stimuli and the activity of intrinsic motivational systems [24]. Nevertheless, the dependency of initial US processing on evaluative conditioning implies Pavlovian conditioned responses should also depend on the motivational and affective associations of conditioned stimuli. This has, in fact, been demonstrated many times and so it should come as no surprise that numerous authors have suggested that Pavlovian CSs can acquire incentive properties [12]. For example, Konorski [8] suggested Pavlovian conditioning comes in two forms: *consummatory* and *preparatory* [25]. In consummatory conditioning (and like evaluative conditioning) the CR often reflects the specific sensory properties of the US; e.g. when a CS paired with food elicits salivation, licking or chewing [26]. In contrast, preparatory conditioning reflects associations between the CS and affective processes activated by the US. As a consequence, preparatory responses are quite general; e.g. CSs paired with appetitive USs (food, water, etc.) often come to elicit approach whereas those paired with aversive USs (footshock or eyeshock) elicit withdrawal [27,28]. This division has also been proposed using other terms; for example in the taxonomy of Berridge and colleagues, consummatory reflexes, such as the orofacial responses to specific tastes and stimuli predicting those tastes, have been called ‘liking’ responses, whereas preparatory responses, such as those elicited by the general appetitive predictions of cues, have been called ‘wanting’ responses [29].

**Figure 3.**
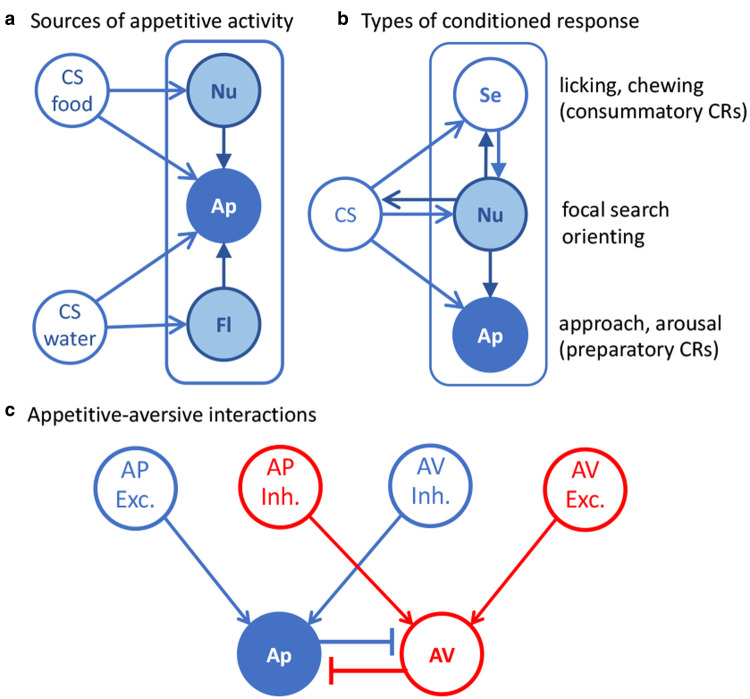
The structure of Pavlovian conditioning and resulting conditioned responses. (**a**) Activation of the appetitive system can emerge both directly and indirectly via the association of sensory events with motivational and affective components of appetitive USs; e.g. between CSs associated with food and water as nutritive (Nu) or fluidic (Fl) events or between these same CSs and a common appetitive affective structure (AP). (**b**) As a consequence of these associations specific conditioned responses can emerge involving orienting, consummatory and preparatory CRs with performance modulated by the central motivational state. (**c**) Conditioned excitors (Exc) and inhibitors (Inh) of different affective class can interact via structural inhibition between appetitive (AP) and aversive (AV) affective systems. For example, an appetitive inhibitor reduces the appetitive activity induced by an appetitive excitor via its activation of the aversive affective system, and vice versa.

The implications of this account for the structure of Pavlovian conditioning are added to that of evaluative conditioning in Figure 3. As shown in Figure 3a, different USs from the same affective class are proposed to activate a common affective system. Further evidence for this claim can be drawn from demonstrations of transreinforcer blocking. Blocking refers to the observation that pretraining one CS often reduces conditioning to a second CS when a compound of these two stimuli is subsequently paired with the US [30]; the pretrained CS is said to block conditioning to the added CS. Whereas the standard blocking procedure employs the same US during pretraining and compound training, Ganesan and Pearce [31] pretrained a CS with a water US before giving compound training with a food US (or vice versa). Conditioned approach to the site of food delivery during the added CS was attenuated by pretraining indicating that prior learning with the water US blocked conditioning to the added CS when paired with food. These results suggest that the positive valence system must feature a common affective structure that can elicit, upon activation, appetitive preparatory responses that are not specific to the US or central motivational state. Similar results have been observed with aversive USs, indicating a common structure for aversive affect [32].

It is clear, however, that associations with motivational states (e.g. associations with nutrient, or fluidic structures) can also modulate preparatory conditioning [26,33] — Figure 3b. Such associations clearly contribute to second-order conditioning [34] and even to the effects of mere exposure to cues in latent inhibition [35]. Furthermore, despite evidence for transreinforcer blocking, and so for the claim that competition between cues reflects competition for association with an affective process, there is also evidence that such competition is modulated by specific central motivational states; e.g. that nutrient predictions compete more strongly with other nutrient predictions than with fluidic ones depending on the prevailing deprivation conditions [36]. Further evidence for the influence of distinct motivational states on preparatory conditioning comes from the literature documenting irrelevant incentive effects [12,37]. For example, Pavlovian CSs, whether discrete stimuli or contexts, associated with a nutritive fluid under food deprivation can motivate performance when the animal is later water deprived [38]. Similarly, stimuli associated with a salty solution under thirst can later motivate performance under a sodium appetite [39,40].

There is also good evidence that this competition between motivational states reflects direct inhibitory connections between them [33,41,42] as there is for inhibitory associations between appetitive and aversive affective systems — i.e. between those processes most commonly argued to produce positive and negative valence [27,43]. This latter evidence comes from studies of appetitive–aversive interaction — Figure 3c. For example, counterconditioning experiments, in which a previously established predictor of an aversive US is subsequently paired with an appetitive US or vice versa, commonly find that rather than enhancing the performance of the previously conditioned CR, this treatment strongly attenuates it, demonstrating that CSs of one affective class inhibit responses controlled by the other affective class [43]. Importantly, this opponent relationship also offers a straight-forward account of the properties of conditioned inhibitors. It has long been known that an inhibitory CS of one affective class has properties in common with an excitatory CS of the opposite affective class. Thus, a CS paired with the omission of expected food is aversive; rats will readily learn to escape from it [44]. Moreover, using a transreinforcer blocking assay, conditioned excitors and inhibitors of opposite affective classes have been found to engage a common incentive process; i.e. in rats a CS that predicts the omission of a food US (i.e. an appetitive inhibitor) blocks aversive conditioning with a shock US, whereas an aversive inhibitor blocks conditioning to a food US [27,45,46].

Clearly, therefore, the study of conditioned preparatory responses reveals multiple routes by which a positive valence system can influence behaviour, whether through specific central motivational states or a more general structure processing common affective information. Furthermore, when taken together with evaluative responses, we can see a hierarchical structure emerging for predictive appetitive learning that, as we will outline next, is critical for supporting perhaps the most important form of flexible adaptive behaviour; that relating to voluntary or goal-directed action.

## Goal-directed actions

Considerable evidence suggests that, in instrumental conditioning, rodents, primates and some birds can encode the relationship between actions and their consequences or outcomes and are extremely sensitive to changes in both the contingency between the performance of an action and the probability of outcome delivery and in the value of the outcome [12,37,47–49]. One of the most striking features of instrumental performance is that, in marked contrast with the Pavlovian CR, it is not *directly* sensitive to shifts in primary motivation; e.g. rats trained hungry to lever press and chain pull with one action earning food pellets and the other a liquid sucrose solution and subsequently shifted to water deprivation only increased their performance of the response trained with the liquid sucrose if they were first allowed to drink the sucrose when thirsty [50]. The shift to water deprivation did not directly impact performance. The same has been observed after many other post-training shifts in motivation. For example, rats trained to lever press for food when food deprived do not immediately reduce their performance on the lever when they are suddenly shifted to an undeprived state; nor do they increase their performance immediately if they are trained undeprived and then given a test on the levers when food deprived [51–53]. In both of these situations, rats only modify their performance after they have been allowed the opportunity to consume the specific food outcome in the test motivational state.

The need for consummatory contact with the instrumental outcome for a shift in primary motivation to affect instrumental performance has also been found in (i) taste aversion-induced devaluation effects [54]; (ii) specific satiety-induced devaluation [55]; (iii) post-training shifts in water deprivation [56]; (iv) changes in outcome value mediated by drug states [53]; and (iv) changes in the value of thermoregulatory [57] and sexual [58] rewards [12,37,49]. In these cases, it is clear that, after a shift in the primary motivational state, rats have to learn about the effect of this shift on the incentive value of an instrumental outcome through consummatory contact before the change will act to affect instrumental performance. This form of learning is referred to as *instrumental incentive learning* [37,49].

**Figure 4.**
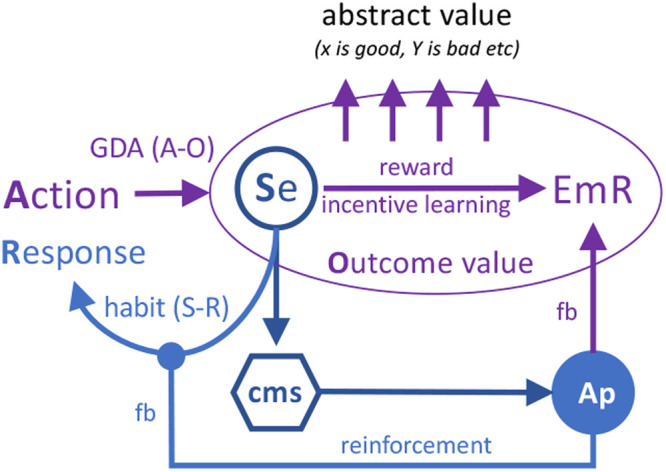
The relationship between reinforcement and reward. The ability of sensory events to evoke activity in the central motivational state and, consequently, affective arousal results in the parsing of appetitive events into two forms of feedback (fb): a reinforcement signal, that strengthens S–R habits, and an emotional response (EmR) that essentially externalises motivational and affective processing. The emotional response is essential for instrumental incentive learning, the process that mediates the reward learning necessary for goal-directed action (GDA). This learning is generated by the relationship between a specific outcome and an emotional response during consummatory experience to generate outcome value. As a consequence of this learning, reward can become more abstract and apparently distinct of its motivational/affective/emotional underpinnings.

These findings suggest that, unlike Pavlovian conditioning, the representation of the instrumental outcome is not directly connected with motivational/affective structures. Rather it is indirectly related via a connection with the emotional feedback induced by the activation of central motivational states; Figure 4. As a consequence, the performance of actions is not affected by shifts in deprivation until the outcome is revalued though consummatory contact in the prevailing motivational state. This suggests that instrumental incentive learning is mediated by an association between the sensory features of the instrumental outcome and emotional feedback driven by the same motivational/affective processes that are engaged by evaluative and Pavlovian incentives [59]. Instrumental incentive learning, although dependent on these processes, involves a distinct associative connection with this emotional response. By establishing the relative hedonic impact of the instrumental outcome, instrumental incentive learning allows animals to encode the reward or incentive value of the consequences of specific actions and so plays a critical role in action selection [47].

## Habits

The motivational control of goal-directed action depends, therefore, on the positive valence processes that mediate Pavlovian evaluative associations through their role in generating emotional feedback via the general affective structures. It is this emotional component that differentiates reward from reinforcement; whereas the former provides the basis for goal-directed action, allowing the experienced value of the outcome to control adaptive behaviour based on current needs or desires, the latter provides the basis for habitual action. However, reinforcement signals, while being generated by the same affective structures, do not rely on emotional feedback to produce their effects on behaviour. Rather, such signals serve merely to ‘stamp in' or serve as a catalyst for the association between a specific motor movement, as a response, and particular stimuli or contexts, giving rise to a form of acquired reflex or habit [60].

Habitual responses are those that have been acquired over the course of training and subsequent over-training. They reflect the development of specific S–R associations strengthened through the selective application of a reinforcement signal [61] — Figure 4. The nature of this signal has been much debated. As described above, one source could lie in drive reduction. However, the reinforcement signal mediating habits is perhaps best characterised as an appetitive or affective signal generated by the positive valence system. There are many such outputs and not all of them will be best understood as reflecting reinforcement *per se*: appetitive arousal and emotional responses, the experience of which confers reward value on the goals of goal-directed actions, do not appear to function as reinforcers although these signals may well correlate with the occurrence of a reinforcement signal [62]. If, however, positive (or negative) reinforcement is just one effect of affective output — where excitatory influences on the appetitive affective system generate positive reinforcement and inhibitory influences on the aversive affective system reflect negative reinforcement — then structures that mediate this output should be involved in multiple coincident effects. In some instances this appears to be true; e.g. structures in the brain that regulate habit acquisition also influence positive (and negative) Pavlovian-instrumental transfer effects [63–65].

Unlike goal-directed actions which are constructed via emotional feedback and incentive learning, habits are motivationally controlled or reinforced by a general affective signal and may share some features with Pavlovian preparatory CRs; e.g. they are directly modulated by changes in drive/motivational state [66].

## The positive valence system

**Figure 5.**
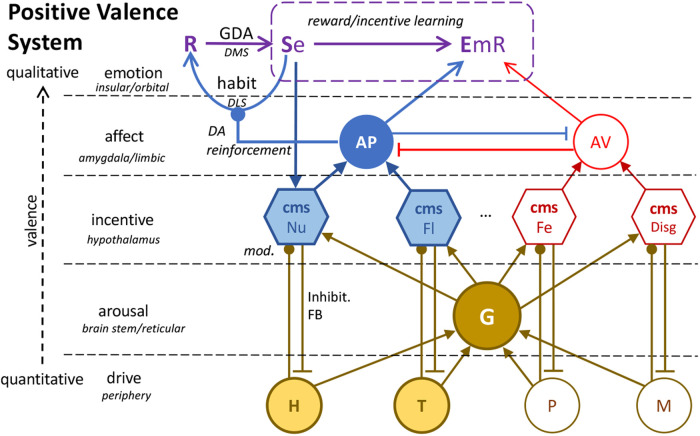
The positive valence system. The combined system showing the integration of evaluative, Pavlovian and instrumental incentive processes mediating the various forms of adaptive behaviour controlled by these processes. This figure shows the levels of processing linking peripheral drive, arousal, incentive, affective and emotional processes and their neural bases. Example central motivational states (cms) are shown tuned to detecting nutrient (Nu), fluid (Fl), threat (Fe) and disgust (Disg) inducing events modulated by peripheral signals for hunger (H), thirst (T), pain (P) and malaise (M); a small subset of likely states. The latter sources of modulation produce general arousal (G) in a reticular arousal system that drives specific and more general classes of motivational, affective and emotional behaviour. DA = dopamine; DMS = dorsomedial striatum; DLS = dorsolateral striatum.

In this brief overview we have attempted to present a general schema revealing the way the Positive Valence System functions to generate the primary classes of adaptive behaviour; i.e. consummatory, preparatory, goal-directed and habitual responses. We outline a hierarchical framework through which the determinants of these behavioural processes are integrated structurally (Figure 5). Nevertheless, in line with the importance of distinguishing between structure and function, it should be noted that this hierarchical structure is not emulated in a hierarchy of function. Associations between sensory events and central motivational states give rise to evaluative conditioned responses but these associations aren't necessary for Pavlovian conditioning. Associations between environmental events can be acquired irrespective their motivational properties, as in sensory preconditioning [67], although whether the influence of associations between purely sensory events is necessarily regulated by motivational processes remains an open question. Likewise, and perhaps the strongest evidence for functional discontinuity, instrumental conditioning does not require, indeed appears actively to compete with, Pavlovian conditioning [68]. It is clear that they are not only mediated by distinct learning rules but also involve distinct value processes: whereas Pavlovian CRs are directly sensitive to shifts in motivation, goal-directed actions — those actions that, in evolutionary terms, critically depend on the development of reward learning [69] — are not immediately affected by shifts in motivational state and rely on the effect of those shifts to become explicit through consummatory contact before they influence performance. Finally, and similarly, goal-directed and habitual instrumental actions also appear to be independent processes, sometimes competing, sometimes collaborating but without one form of action control depending on the other [60]. It is these discontinuities that render attempts to generate a common syntax for functional behavioural systems, e.g. in active inference accounts, so unconvincing [70–72].

The hierarchical structure of the positive valence system is implemented within a similarly hierarchically integrated system of neural structures (Figure 5). Peripheral signalling induced by changes associated with deprivation both alter the activity of arousal-related structures in the reticular activating system and modulate hypothalamic nuclei associated with specific central motivational states [73], that together engage the evaluative incentive learning processes necessary for sensory events to become represented as USs. Connections of these ascending inputs to limbic sensory and affective structures provide the basis for consummatory and preparatory Pavlovian conditioning, that are dissociated neurally, even within the same amygdala complex: consummatory conditioning relying on cortico-limbic-hypothalamic network centred on the basolateral amygdala (BLA) and preparatory conditioning on a parallel network centred on the amygdala central nucleus (CeA), rather than the BLA [74–77]. These networks have in common a close association with midbrain dopamine neurons, albeit with distinct populations in the ventral tegmental area and substantia nigra, respectively. Interestingly, a relation between affective processes in CeA and the lateral substantia nigra [78] appears to provide a structural connection between the CeN and the dorsolateral striatum [79], providing the dopamine-related reinforcement signal that regulates the acquisition of habits [47,80]. Finally, these ascending midbrain-limbic networks give rise to the emotional processes supporting instrumental incentive learning, the latter supported neurally by a reward network connecting the BLA, insular cortex [81–83] and ascending connections with orbitofrontal and cingulate cortical regions. These structures, encoding reward values, are integrated with an action network centred on the striatum, encoding action-outcome associations, to control goal-directed performance [84]; a limbic-motor interface, brought to prominence by Mogenson et al. [85].

This integrated structure provides a mapping from fundamental regulatory to higher-order motivational and emotional processes that ensures behaviour remains broadly adaptive, however, flexible and tenuous its links to primary motivational states become. This hierarchical view provides, therefore, a solution to the problem of providing a deterministic account of higher order or abstract goals. However, mapping the appetitive valence system onto a precise neural architecture may not only be useful for understanding the structural basis of behaviour; it can also help to pinpoint the source of abnormal neural-behavioural activity associated with specific psychiatric disorders. Although it is well-established that aberrations in motivational control are observed in patients with disorders such as schizophrenia, depression, OCD and addiction [86], exposing the precise structural processes that underlie such symptoms may provide a route for developing more targeted therapies. There are various other open questions: e.g. the many gaps in our understanding of the interaction between peripheral and central nervous systems; how peripheral regulatory process affect complex brainstem and hypothalamic nuclei to influence motivational control processes; how a system composed of multiple distinct central motive states influence cognitive networks; and whether motivational control of higher order, metacognitive and conscious states are similarly organised [69]. Addressing these questions will go a long way towards a fully deterministic account of motivational control generally and of the positive valence system in particular.

## Summary

- The positive valence system is hierarchically structured but produces classes of adaptive behaviour that either compete or are functionally independent.
- Stimulus connections with central motivational states produce an associative complex that allows those stimuli to elicit unconditioned responses.
- Associations between stimulus events and stimulus-motivational state complexes produce both consummatory and preparatory Pavlovian conditioned responses.
- Pavlovian preparatory responses are driven by associations with the appetitive affective system eliciting conditioned approach, general search and affective feedback used as a reinforcement signal to acquire stimulus–response habits
- Appetitive affective activity also evokes emotional responses with which sensory events can become associated to generate the experienced values assigned to the goals of goal-directed actions.

## Abbreviations

BLA: basolateral amygdala
CeA: central nucleus
cms: central motivational state
CR: conditioned responses
UR: unconditioned response
USs: unconditioned stimuli
